# Comment on “Reflections on patient engagement by patient partners: How it can go wrong”

**DOI:** 10.1186/s40900-023-00534-2

**Published:** 2023-12-22

**Authors:** Paola Zaratin, Usman Khan, Guendalina Graffigna

**Affiliations:** 1 Italian Multiple Sclerosis Society Foundation, Genoa, Italy; 2 Institute for Health Care Policy, KU Louvain, Leuven, Belgium; 3 Department of Psychology, EngageMinds HUB - Consumer, Food & Health Engagement Research Center, Università Cattolica del Sacro Cuore, Milan, Italy

**Keywords:** Patient engagement, tokenism, Participatory governance model, Responsible research innovation

## Abstract

As patient-advocacy, public policy and clinical researchers with special knowledge on Responsible Research Innovation (RRI) governance and the public health and psychology underlying patient engagement, we read with interest the comment contribution by Richards et al., “Reflections on patient engagement by patient partners: How it can go wrong” (Richards et al. in Res Involv Engagem 9:41, 2023. 10.1186/s40900-023-00454-13). As a way to help meet the “take-away actions for readers” included by the authors at the end of the article, we would like to further stimulate discussion with relevant stakeholder communities about the need to rethink the use of “expert patient”. Based on our experience, the lack of a governance model engaging patients who are representative of the target patient community, as opposed to expert patients, is at the root of the tokenistic approach, the “patient partner as a checkmark statement” and the “lack of recognizing the vulnerability of patient partners”, which results in “patient engagement going wrong”. According to our experience, the Responsible Research Innovation (RRI) MULTI-ACT model has the potential to help meet these challenges.

## Plain Language Abstract

In their article, Richards et al. (Res Involv Engagem 9:41, 2023. 10.1186/s40900-023-00454-13) reflect on the practices of patient engagement in research, highlighting not only the value and opportunity of including people suffering from a disease as coinvestigators in the research process but also the risks and errors that must be avoided in such a process. Along this line of discussion—and based on our experience of research, teaching and patient advocacy—we further reflect on the nature of the concept of “expert patient” in this paper. In particular, we argue for the importance of guaranteeing the wide inclusion of all the different kinds of “experiential knowledge” that patients may bring into the research project, and we question the risk of a poorly representative approach if only guided to select patients with acquired knowledge and expertise about the scientific research process itself. Based on our experience, the Responsible Research Innovation (RRI) governance models have the potential to help meet these challenges.

## Addressing takeaway action for readers from the Canadian paper: *from “expert patients” to science of patients’ engagement: A call to action to relevant stakeholders*

The term “expert patient” has a long history in health care [2, 3], and over the past decade, it has emerged as an important way to help improve the relevance, quality, and impact of clinical research. Indeed, much of the current guidelines for patient engagement focus on enabling “expert patients” in the “medicine life cycle” [4]. In our experience, the individual contribution of a single expert patient, although trained [5], does not represent the collective contribution of the relevant community from research to care (regardless of whether treatments, medical devices, or other sectors are being discussed). Furthermore, this sounds like a tokenistic approach to revising scientific research processes. Even if the proposed goal is to innovate these processes to include more of patients’ experiential knowledge, the approaches for reaching this goal do not seem to include any consideration of the governance and psychosocial requirements for achieving this in a meaningful way. We acknowledge that ensuring collective contributions from research to care requires a dedicated governance model and resources [6]. What began as an extension of patient advocacy [7] must now evolve into the new discipline of science of patient engagement [8] aimed at understanding and incorporating patient experiences, needs, expectations, and perspectives into the process of health research and care. Capturing the patient voice and making it scientifically relevant for other stakeholders will provide the aspect of validity to patient engagement [9] and thus avoid considerations of “patient partners as a check mark”. Achieving this ambitious goal relies on our ability to meet representativeness of the relevant patients’ experiential knowledge and to question consolidated processes of research management [10]. In our view, an effort to meet representativeness should focus more on identifying individuals with a broad range of experiential knowledge of living with the disease and characterizing their level of engagement based on other relevant factors, including education and economic and social characteristics [11], thus recognizing and valuing the vulnerability of patient partners. Patients may be at different stages of their psychological process of engagement: some may be too psychologically burdened to participate, even though their needs or experiences may be of crucial importance for orienting future research and development in medicine. Currently, risk is a sort of “self-selection bias” in the inclusion of patients in the research process as they are “top-down” designed and too oriented by expert and scientific knowledge and expertise. This paradoxically enables an unequal process of patient engagement, which risks to bring biases in the scientific process rather than making it more inclusive and more impactful. As a consequence, the expert patient may seem to provide easy solutions for training patients in specific proficiency areas (e.g., including the life cycle of medicines, clinical trial development, databases and biobanking generation, artificial intelligence and digital transformation). This results in transforming them into little scientists who can adhere to researchers’ expectations and prejudices about patients’ needs instead of deeply questioning research procedures and formats to make them able to include patients’ experiential knowledge. Patients’ experiential knowledge (aka the lived experience of patients) is now widely recognized to be suited to complement the expertise of researchers. Following this, we should aim for patients skilled in sharing their experiential knowledge rather than for expert patients in proficiency areas that are helpful for scientists. Thinking about the digital transformation that awaits us, a very specialized proficiency area, it is important to be guided by patients’ wisdom for innovation to be constructive and not destructive [12] rather than to ask them to develop artificial intelligence algorithms. Shifting away from relying on a few expert patients and working towards engaging a broader community through the science of patients’ engagement is the shared responsibility of all the stakeholders, and it is a foundational Responsible Research Innovation (RRI) skill [13] for meeting equity, diversity and inclusion needs [14]. Our definition of science of patient engagement comes from the Responsible Research Innovation (RRI) EU MULTI-ACT project (A Collective Research Impact Framework and multivariate models to foster the true engagement of actors and stakeholders in Health Research and Innovation) [8]. According to the European RRI portfolio and based on our experience[15], the MULTI-ACT model has the potential to enable institutional changes [8, 16] for applying multistakeholder participatory governance in patient engagement in health research [9]. The MULTI-ACT patients’ engagement is not a stand-alone strategy but it is empowered by the other two components of the model (i.e. governance criteria and multidisciplinary impact assessment), allowing all the stakeholders to acknowledge the value of science with and of patient input, and to align their interests with those of the patients, towards a common mission and shared agenda. (Table 1).

**Table 1:** The MULTI-ACT patients’ engagement model guiding principles

The added values of the MULTI-ACT patients’ engagement model
Mission and agenda-driven engagement model to incorporate patients' input and experience in research steps where their contributions can increase the impact of health research initiatives
Set up an Engagement Coordination Team (ECT), including people living with the disease and their caregivers, that is in charge to ensure the engagement of a community that is representative to meet a given research mission and agenda
A governance model that integrates heterogeneity in perspectives (meeting equity, diversity, and inclusion) by shifting away from relying on a few expert patients and work towards engaging a broader patients' community through various research methods
People living with the disease and their caregivers do not need to be scientific experts to participate and contribute. The real value is not their scientific expertise, but their experiential knowledge
Identify key indicators to be used for assessing the return on engagement and to monitor if patient engagement has reached the expected impact on the initiatives

**Fig. 1:**
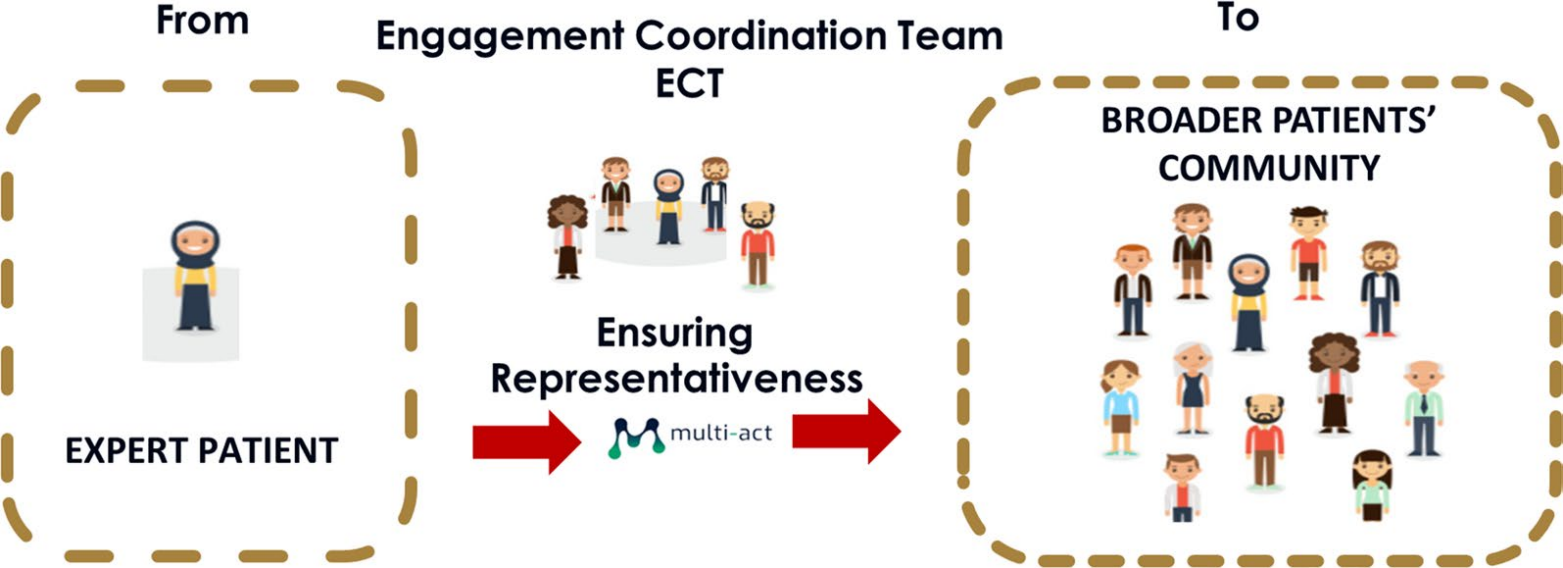
Ensuring Representativeness through the MULTI-ACT Engagement Coordination Team governance body (@MULTI-ACT-FISM COPYRIGHTS)

The model introduced the concept of the Engagement Coordination Team (ECT) as a patient engagement governance body. Thus, patients who are members of the ECT are in charge of ensuring the engagement of a community that is representative of a given research mission and agenda (Fig. 1.). To achieve this, the ECT requires key skills, driven by the sensible understanding of clinical and psychosocial implications.

Importantly, the MULTI-ACT model aims to meet “representativeness” targets for an engagement that is distinct from statistical sampling in that it focuses more on identifying individuals with similar experiential knowledge of living with the disease rather than meeting a known statistical threshold for the number of patient participants [17]. The evaluation of patient engagement experience itself may guarantee the adequate representativeness of patients included in such consultative initiatives [18]. Patient availability to engage in health research and care may vary depending on demographic characteristics, phase of the clinical pathway, psychological factors, comorbidities, place of residence, level of family and caregiver support, literacy, culture, and goals of care. In this context, measuring levels of patient involvement will guarantee the personalization of participation offered to different patient targets and will guarantee a broader scope of participation, even for those representatives who are apparently difficult to reach and facing difficulty but whose experiential knowledge and whose inputs are also extremely important. Patients' engagement, indeed, should be seen also in the light of people psychological readiness to get involved in health research and care, based on their peculiar illness experience. In other terms, guarantee a good representativeness of patients engaged in research means guarantee equal access to all the different illness experiences which may be relevant for the purposes of the engagement activity itself. In their article, Richards et al. [1] highlighted tokenism and lack of recognition of the vulnerability of patient partners among the main errors that must be avoided to ensure meaningful patient engagement. As described in this contribution, we believe that ensuring patient representativeness clearly falls within the governance aspects that will help address the above challenges and provide content of validity (and clinical soundness) to a more rigorous science of patients' engagement.

## Data Availability

Not applicable.
